# Supplementary material for: A dynamic neural network model for predicting risk of Zika in real time
Source: BMC Med. 2019 Sep 2;17:171. doi: 10.1186/s12916-019-1389-3 (PMC6717993; doi:10.1186/s12916-019-1389-3)
Supplement: Supplementary file 13 — Table S2. Summary of model performance. ACC is presented for each combination of risk indicator (case count and incidence rate), classification scheme (i.e., R = 0.1, 0.2, 0.3, 0.4, 0.5, and A = 90, 80, 70, 60, 50) and forecast window (i.e., N = 1, 2, 4, 8, and 12), for selected Epi weeks throughout the epidemic. ROC AUC (averaged over all locations and all EPI weeks) is computed for all combinations of risk indicator (case count and incidence rate), classification scheme (i.e., R = 0.1, 0.2, 0.3, 0.4, 0.5 and A = 90, 80, 70, 60, 50) and forecast window (i.e., N = 1, 2, 4, 8 and 12). (DOCX 21 kb) [file 12916_2019_1389_MOESM13_ESM.docx]

Table S2. Summary of model performance

| **Risk Indicator** | **Risk Classification** | | **Prediction Window Size (*N* in weeks)** | **Model Performance** | | | | | | |
| --- | --- | --- | --- | --- | --- | --- | --- | --- | --- | --- |
|  |  |  |  | Area Under ROC Curve (AUC) | Prediction Accuracy (ACC) | | | | | |
|  |  |  |  |  | Overall  Avg. | Selected Epidemiological Weeks | | | | |
|  | Type | Scheme |  |  |  |  |  |  |  |  |
|  |  |  |  |  |  | 30 | 40 | 50 | 60 | 70 |
| Incidence Rate | Relative (R, top fraction as high risk) | 0.1 | 1 | 0.92 | 95.71 | 90.57 | 98.11 | 94.34 | 94.34 | 96.23 |
|  |  |  | 2 | 0.89 | 94.29 | 92.45 | 88.68 | 90.57 | 92.45 | 92.45 |
|  |  |  | 4 | 0.80 | 91.30 | 86.79 | 94.34 | 94.34 | 92.45 | 88.68 |
|  |  |  | 8 | 0.70 | 86.34 | 84.91 | 86.79 | 88.68 | 81.13 | 86.79 |
|  |  |  | 12 | 0.57 | 82.57 | 84.91 | 83.02 | 81.13 | 81.13 | 81.13 |
|  |  | 0.2 | 1 | 0.92 | 93.07 | 81.13 | 98.11 | 94.34 | 94.34 | 83.02 |
|  |  |  | 2 | 0.89 | 90.00 | 84.91 | 92.45 | 88.68 | 92.45 | 94.34 |
|  |  |  | 4 | 0.78 | 84.68 | 75.47 | 86.79 | 88.68 | 86.79 | 79.25 |
|  |  |  | 8 | 0.62 | 75.22 | 75.47 | 84.91 | 81.13 | 64.15 | 67.92 |
|  |  |  | 12 | 0.54 | 68.96 | 79.25 | 73.58 | 83.02 | 62.26 | 73.58 |
|  |  | 0.3 | 1 | 0.92 | 90.70 | 83.02 | 94.34 | 94.34 | 83.02 | 94.34 |
|  |  |  | 2 | 0.87 | 86.74 | 83.02 | 92.45 | 81.13 | 83.02 | 81.13 |
|  |  |  | 4 | 0.80 | 80.85 | 62.26 | 92.45 | 83.02 | 79.25 | 69.81 |
|  |  |  | 8 | 0.65 | 70.10 | 66.04 | 67.92 | 79.25 | 58.49 | 66.04 |
|  |  |  | 12 | 0.57 | 63.37 | 66.04 | 67.92 | 64.15 | 60.38 | 62.26 |
|  |  | 0.4 | 1 | 0.93 | 90.46 | 83.02 | 92.45 | 94.34 | 83.02 | 83.02 |
|  |  |  | 2 | 0.89 | 86.79 | 77.36 | 90.57 | 86.79 | 84.91 | 81.13 |
|  |  |  | 4 | 0.80 | 79.36 | 62.26 | 79.25 | 81.13 | 71.70 | 66.04 |
|  |  |  | 8 | 0.68 | 68.47 | 60.38 | 75.47 | 77.36 | 62.26 | 67.92 |
|  |  |  | 12 | 0.56 | 59.82 | 60.38 | 66.04 | 64.15 | 39.62 | 58.49 |
|  |  | 0.5 | 1 | 0.93 | 89.51 | 75.47 | 92.45 | 83.02 | 90.57 | 86.79 |
|  |  |  | 2 | 0.90 | 86.21 | 84.91 | 84.91 | 86.79 | 77.36 | 71.70 |
|  |  |  | 4 | 0.83 | 77.67 | 56.60 | 90.57 | 81.13 | 62.26 | 67.92 |
|  |  |  | 8 | 0.73 | 66.42 | 56.60 | 75.47 | 73.58 | 49.06 | 62.26 |
|  |  |  | 12 | 0.60 | 56.16 | 56.60 | 58.49 | 56.60 | 50.94 | 52.83 |
| Case Counts |  | 0.1 | 1 | 0.95 | 96.95 | 100 | 94.34 | 98.11 | 92.45 | 96.23 |
|  |  |  | 2 | 0.92 | 96.12 | 94.34 | 92.45 | 96.23 | 96.23 | 92.45 |
|  |  |  | 4 | 0.85 | 93.13 | 90.57 | 98.11 | 88.68 | 88.68 | 86.79 |
|  |  |  | 8 | 0.75 | 90.63 | 92.45 | 90.57 | 94.34 | 81.13 | 88.68 |
|  |  |  | 12 | 0.66 | 87.05 | 90.57 | 90.57 | 90.57 | 79.25 | 88.68 |
|  |  | 0.2 | 1 | 0.93 | 93.54 | 92.45 | 94.34 | 94.34 | 94.34 | 90.57 |
|  |  |  | 2 | 0.90 | 92.27 | 92.45 | 92.45 | 90.57 | 84.91 | 92.45 |
|  |  |  | 4 | 0.84 | 88.09 | 84.91 | 94.34 | 94.34 | 79.25 | 81.13 |
|  |  |  | 8 | 0.73 | 81.87 | 84.91 | 81.13 | 86.79 | 77.36 | 79.24 |
|  |  |  | 12 | 0.68 | 78.25 | 84.91 | 77.36 | 84.91 | 77.36 | 84.91 |
|  |  | 0.3 | 1 | 0.94 | 93.41 | 92.45 | 96.23 | 86.79 | 94.34 | 86.79 |
|  |  |  | 2 | 0.90 | 89.82 | 88.68 | 90.57 | 88.68 | 79.25 | 94.34 |
|  |  |  | 4 | 0.84 | 84.31 | 73.58 | 90.57 | 83.02 | 71.70 | 86.79 |
|  |  |  | 8 | 0.71 | 76.46 | 71.70 | 83.02 | 88.68 | 62.26 | 75.47 |
|  |  |  | 12 | 0.64 | 71.66 | 73.58 | 73.58 | 69.81 | 67.92 | 69.81 |
|  |  | 0.4 | 1 | 0.94 | 91.68 | 86.79 | 90.57 | 96.22 | 81.13 | 83.02 |
|  |  |  | 2 | 0.91 | 88.52 | 90.57 | 90.57 | 90.57 | 84.91 | 86.79 |
|  |  |  | 4 | 0.83 | 81.67 | 69.81 | 83.02 | 88.68 | 73.58 | 79.25 |
|  |  |  | 8 | 0.73 | 72.85 | 69.81 | 79.25 | 83.02 | 60.38 | 75.47 |
|  |  |  | 12 | 0.64 | 65.22 | 67.92 | 62.26 | 73.58 | 62.26 | 75.47 |
|  |  | 0.5 | 1 | 0.94 | 91.16 | 81.13 | 86.79 | 92.45 | 92.45 | 84.91 |
|  |  |  | 2 | 0.91 | 86.90 | 83.02 | 94.34 | 77.36 | 79.25 | 86.79 |
|  |  |  | 4 | 0.84 | 78.46 | 56.60 | 88.68 | 79.25 | 73.58 | 71.70 |
|  |  |  | 8 | 0.74 | 68.05 | 54.72 | 79.25 | 69.81 | 52.83 | 69.81 |
|  |  |  | 12 | 0.62 | 58.31 | 58.49 | 52.83 | 71.70 | 54.72 | 56.60 |
| Incidence Rate | Absolute (A, avg. of incidence rate percentile) | 90 | 1 | 0.95 | 97.57 | 100 | 96.23 | 100 | 96.23 | 94.34 |
|  |  |  | 2 | 0.93 | 96.55 | 96.23 | 96.23 | 100 | 90.57 | 96.23 |
|  |  |  | 4 | 0.85 | 94.26 | 92.45 | 92.45 | 94.34 | 83.02 | 92.45 |
|  |  |  | 8 | 0.71 | 90.66 | 92.45 | 92.45 | 96.23 | 77.36 | 92.45 |
|  |  |  | 12 | 0.62 | 88.01 | 92.45 | 88.68 | 90.57 | 83.02 | 84.91 |
|  |  | 80 | 1 | 0.93 | 94.42 | 90.57 | 98.11 | 88.68 | 83.02 | 88.68 |
|  |  |  | 2 | 0.90 | 92.32 | 84.91 | 94.34 | 90.57 | 90.57 | 83.02 |
|  |  |  | 4 | 0.82 | 87.75 | 77.36 | 86.79 | 83.02 | 83.02 | 75.47 |
|  |  |  | 8 | 0.68 | 80.30 | 77.36 | 77.36 | 75.47 | 64.15 | 71.70 |
|  |  |  | 12 | 0.58 | 75.55 | 77.36 | 77.36 | 73.58 | 66.04 | 81.13 |
|  |  | 70 | 1 | 0.94 | 92.32 | 84.91 | 92.45 | 90.57 | 90.57 | 88.68 |
|  |  |  | 2 | 0.91 | 90.62 | 79.25 | 86.79 | 86.79 | 86.79 | 86.79 |
|  |  |  | 4 | 0.84 | 84.23 | 69.81 | 86.79 | 88.68 | 71.70 | 67.92 |
|  |  |  | 8 | 0.71 | 75.54 | 67.92 | 83.02 | 84.91 | 58.49 | 71.70 |
|  |  |  | 12 | 0.62 | 68.74 | 66.04 | 79.25 | 69.81 | 52.83 | 67.92 |
|  |  | 60 | 1 | 0.94 | 92.50 | 81.13 | 92.45 | 84.91 | 90.57 | 90.57 |
|  |  |  | 2 | 0.92 | 88.97 | 86.79 | 90.57 | 86.79 | 88.68 | 79.25 |
|  |  |  | 4 | 0.85 | 82.17 | 66.04 | 86.79 | 81.13 | 67.92 | 79.25 |
|  |  |  | 8 | 0.72 | 71.86 | 62.26 | 75.47 | 75.47 | 56.60 | 58.49 |
|  |  |  | 12 | 0.61 | 61.49 | 62.26 | 66.04 | 67.92 | 47.17 | 60.38 |
|  |  | 50 | 1 | 0.94 | 91.65 | 81.13 | 94.34 | 84.91 | 88.68 | 77.36 |
|  |  |  | 2 | 0.91 | 87.91 | 79.25 | 94.34 | 77.36 | 77.36 | 79.25 |
|  |  |  | 4 | 0.84 | 80.20 | 56.60 | 90.57 | 73.58 | 67.92 | 69.81 |
|  |  |  | 8 | 0.74 | 68.92 | 52.83 | 77.36 | 73.58 | 56.60 | 67.92 |
|  |  |  | 12 | 0.63 | 56.71 | 50.94 | 60.38 | 60.38 | 39.62 | 49.06 |
