# Supplementary material for: A dynamic neural network model for predicting risk of Zika in real time
Source: BMC Med. 2019 Sep 2;17:171. doi: 10.1186/s12916-019-1389-3 (PMC6717993; doi:10.1186/s12916-019-1389-3)
Supplement: Supplementary file 14 — Figure S2. Aggregate model performance measured by ROC AUC. The ROC AUC is averaged over all locations and all weeks, for each relative risk classification scheme, i.e., R = 0.1, 0.2, 0.3, 0.4, 0.5 and forecast window i.e., N = 1, 2, 4, 8, and 12. For the results shown the risk indicator is case counts. (DOCX 212 kb) [file 12916_2019_1389_MOESM14_ESM.docx]

(a)

(b)

(c)

(d)

(e)

**Figure S2. Aggregate model performance measured by ROC AUC** (averaged over all locations and all weeks) for each (relative) classification scheme, *i.e*., (a) R = 0.1, (b) R = 0.2, (c) R = 0.3, (d) R = 0.4, (e) R = 0.5 and forecast windows (i.e., *N* = 1, 2, 4, 8 and 12), where the risk indicator is case counts.
