# Supplementary material for: A dynamic neural network model for predicting risk of Zika in real time
Source: BMC Med. 2019 Sep 2;17:171. doi: 10.1186/s12916-019-1389-3 (PMC6717993; doi:10.1186/s12916-019-1389-3)
Supplement: Supplementary file 15 — Table S1. Summary of model sensitivity to feature selection. The ACC and ROC AUC performance of the model is computed and presented under different combinations of input data features. The proposed model is compared against two baseline models; one includes only case (and incidence) data, and the second includes case and all non-travel related data, while the final proposed model includes all features. The results presented are for the absolute risk classification scheme, where the risk indicator is incidence rate. (DOCX 21 kb) [file 12916_2019_1389_MOESM15_ESM.docx]

Table S1. Summary of NARX model sensitivity to feature selection

| **Absolute Risk Classification Scheme** | **Prediction Window Size**  (*N* in weeks) | **Baseline Model 1**  (regional case count and incidence rate) | | **Baseline Model 2**  (case data and all non-travel related features*) | | **Proposed Model**  (all features**) | |
| --- | --- | --- | --- | --- | --- | --- | --- |
|  |  | Area Under ROC Curve (AUC) | Overall Prediction Accuracy (ACC) | Area Under ROC Curve (AUC) | Overall Prediction Accuracy (ACC) | Area Under ROC Curve (AUC) | Overall Prediction Accuracy (ACC) |
| A=90 | 1 | 0.94 | 96.92 | 0.92 | 96.77 | 0.96 | 97.57 |
|  | 2 | 0.89 | 95.48 | 0.87 | 95.99 | 0.92 | 96.55 |
|  | 4 | 0.76 | 93.35 | 0.78 | 93.07 | 0.83 | 94.26 |
|  | 8 | 0.66 | 89.51 | 0.66 | 89.96 | 0.69 | 90.66 |
|  | 12 | 0.62 | 88.24 | 0.62 | 87.53 | 0.64 | 88.01 |
| A=80 | 1 | 0.91 | 93.75 | 0.9 | 93.05 | 0.92 | 94.42 |
|  | 2 | 0.86 | 91.42 | 0.86 | 90.81 | 0.89 | 92.32 |
|  | 4 | 0.75 | 86.54 | 0.78 | 86.14 | 0.82 | 87.75 |
|  | 8 | 0.64 | 79.69 | 0.63 | 79.76 | 0.69 | 80.30 |
|  | 12 | 0.56 | 75.36 | 0.56 | 75.40 | 0.6 | 75.55 |
| A=70 | 1 | 0.94 | 92.14 | 0.94 | 92.92 | 0.94 | 92.32 |
|  | 2 | 0.91 | 90.46 | 0.91 | 90.30 | 0.9 | 90.62 |
|  | 4 | 0.83 | 84.34 | 0.84 | 84.65 | 0.85 | 84.23 |
|  | 8 | 0.68 | 75.18 | 0.68 | 75.06 | 0.71 | 75.54 |
|  | 12 | 0.61 | 70.51 | 0.6 | 69.48 | 0.58 | 68.74 |
| A=60 | 1 | 0.94 | 92.76 | 0.95 | 92.12 | 0.94 | 92.50 |
|  | 2 | 0.91 | 88.95 | 0.91 | 89.16 | 0.92 | 88.97 |
|  | 4 | 0.85 | 83.95 | 0.85 | 83.33 | 0.85 | 82.17 |
|  | 8 | 0.73 | 74.42 | 0.73 | 74.54 | 0.73 | 71.86 |
|  | 12 | 0.64 | 65.82 | 0.62 | 65.59 | 0.61 | 61.49 |
| A=50 | 1 | 0.94 | 91.39 | 0.94 | 91.83 | 0.95 | 91.65 |
|  | 2 | 0.91 | 87.70 | 0.9 | 87.43 | 0.92 | 87.91 |
|  | 4 | 0.84 | 82.06 | 0.85 | 82.26 | 0.85 | 80.20 |
|  | 8 | 0.76 | 73.71 | 0.77 | 74.32 | 0.73 | 68.92 |
|  | 12 | 0.61 | 60.90 | 0.6 | 60.30 | 0.62 | 56.71 |

*Feature set includes regional case counts, regional incidence rates, regional suitability, regional GDP, regional physicians, regional hospital beds, regional population density

**Feature set includes regional case counts, regional incidence rates, regional suitability, regional GDP, regional physicians, regional hospital beds, regional population density, weekly incoming travel, weekly outgoing travel, weekly case-weighted travel risk, weekly incidence-weighted travel risk.
